# Towards molecular-pathology informed clinical trials in childhood arthritis to achieve precision medicine in juvenile idiopathic arthritis

**DOI:** 10.1136/ard-2022-222553

**Published:** 2022-12-05

**Authors:** Lucy R Wedderburn, Athimalaipet V Ramanan, Adam P Croft, Kimme L Hyrich, Andrew D Dick, the CLUSTER Consortium

**Affiliations:** 1 UCL GOS Institute of Child Health, University College London, London, UK; 2 Centre for Adolescent Rheumatology Versus Arthritis at UCL UCLH and GOSH, UCL, London, UK; 3 National Institute of Health Research Biomedical Research Centre at GOSH London UK, Great Ormond Street Hospital, London, UK; 4 Department of Paediatric Rheumatology, Bristol Royal Hospital for Children, Bristol, UK; 5 Translational Health Sciences, University of Bristol, Bristol, UK; 6 Rheumatology Research Group, Institute of Inflammation and Ageing, University of Birmingham, Birmingham, UK; 7 National Institute of Health Research Biomedical Research Centre, University Hospitals Birmingham NHS Foundation Trust, Birmingham, UK; 8 Centre for Epidemiology Versus Arthritis, The University of Manchester, Manchester Academic Health Science Centre, Manchester, UK; 9 National Institute of Health Research Manchester Biomedical Research Centre, Manchester University NHS Foundation Trust, Manchester, UK; 10 UCL Institute of Ophthalmology, University College London, London, UK; 11 National Institute of Health Research Biomedical Research Centre, Moorfields and UCL Institute of Ophthalmology, London, UK

**Keywords:** Arthritis, Juvenile, Biological Therapy, Immune System Diseases, Inflammation

## Abstract

In childhood arthritis, collectively known as Juvenile idiopathic arthritis (JIA), the rapid rise of available licensed biological and targeted small molecule treatments in recent years has led to improved outcomes. However, real-world data from multiple countries and registries show that despite a large number of available drugs, many children and young people continue to suffer flares and experience significant periods of time with active disease for many years. More than 50% of young people with JIA require ongoing immune suppression well into adult life, and they may have to try multiple different treatments in that time. There are currently no validated tools with which to select specific treatments, nor biomarkers of response to assist in such choices, therefore, current management uses essentially a trial-and-error approach. A further consequence of recent progress is a reducing pool of available children or young people who are eligible for new trials. In this review we consider how progress towards a molecular based approach to defining treatment targets and informing trial design in JIA, combined with novel approaches to clinical trials, could provide strategies to maximise discovery and progress, in order to move towards precision medicine for children with arthritis.

## Background

The last two decades have seen a remarkable burgeoning of available medications to treat immune mediated inflammatory diseases (IMID). New agents are now used widely when conventional synthetic (cs) disease-modifying antirheumatic drugs (DMARDs) such as methotrexate fail to control disease, including biological (b) DMARDs, and more recently small molecules, also known as targeted synthetic (ts) DMARDs, such as Janus kinase inhibitors.^1–3^ One of the most common IMID of childhood is Juvenile idiopathic arthritis (JIA). JIA is an umbrella term used to describe a set of heterogeneous conditions in which arthritis lasting for at least 6 weeks starts before the age of 16 years.^4^ A recent UK population-based study estimated prevalence to be 43.5 per 100 000 children and young people (CYP), or approximately 1 in 2000 CYP under 16 years of age.^5^

Currently, the underlying pathophysiology, molecular basis and key drivers of disease are unknown, and therefore, to facilitate clinical research, the International League of Associations for Rheumatology (ILAR) classification criteria, which largely group children according to their clinical phenotype, is used.^4^ This classification system has enabled clinical trials, basic and clinical research to use a common nomenclature, to group patients by phenotype and compare cohorts of childhood arthritis. However, the ILAR classification is not without its challenges, including that it is based on clinical features such as number of joints, rather than underlying molecular ‘pathotype’. With some exceptions, it does not map to treatment response or inform best initial treatment for any one group. If treatment of childhood arthritides was aligned to molecular signatures, rather than clinical phenotype alone, this could allow for an evidence-based approach to the use of targeted therapies, permit comparative analyses with the parallel adult conditions, the use of biological outcome measures in clinical trials and be more inclusive for trials by overcoming the exclusion of undifferentiated arthritis group, which is highly heterogeneous.^6–8^ Ultimately, such an approach should improve patient outcomes through better targeting of treatments early in disease.

### Evidence generation for the current treatment of JIA

Standard management of many forms of JIA includes non-steroidal anti-inflammatory medication, intra-articular joint injection and/or first-line csDMARD treatment using methotrexate.^7 9 10^ When these do not adequately control disease, treatment is switched to a bDMARD, typically starting with a tumour necrosis factor-alpha TNFα inhibitor. The advent of TNFα inhibitors and other cytokine blocking therapies for adults with inflammatory arthritis, coupled with the landmark legislation by the US Food and Drug administration and European Medicines Agency making it mandatory for Industry to do trials in children (Best Pharmaceuticals for Children Act in the USA and specific legislation for the development of paediatric medicines (Paediatric Regulation) in the European Union^11–14^ have led to a rapid rise in the number of licensed medicines for children with arthritis. The evidence base for safety and efficacy of these agents in JIA has been made possible by major efforts of international collaborative networks, delivering clinical trials (including the Paediatric Rheumatology Collaborative Study Group, Pediatric Rheumatology International Trials Organization, UK Paediatric Rheumatology Clinical Studies Group and more recently the Childhood Arthritis and Rheumatology Research Alliance).

There have been many interventional trials of b/tsDMARDs in JIA, although most limited their recruitment to certain subtypes (hereafter referred to as categories as in the ILAR criteria), of JIA. Most have used the so-called withdrawal design^15^ where all subjects receive the drug initially in an open-label design, and those who achieve a response, typically the paediatric American College of Rheumatology (ACR)-30,^16^ are then randomised to stop drug and receive placebo, or continue active drug, with the primary outcome being the proportion of children who subsequently flare. Those who did not initially respond are not randomised to this second phase, meaning the randomised phase is limited to responders only. Some of these studies recruited cases with ‘polyarticular course disease’ only, where children included were from several categories of JIA (extended oligoarticular, polyarticular RF− and RF+, systemic JIA (sJIA) with poly course and in some trials, enthesitis-related arthritis, ERA-JIA and psoriatic JIA) with five or more active joints. These studies were highly successful in achieving access to new drugs for children with arthritis. Most showed benefit in a proportion of cases across categories, although were underpowered to stratify by subtype. Patients who did not reach a paediatric ACR30 were in general not studied in detail creating a systematic lost opportunity to understand the underlying causes of non-response in these trials.

Several clinical trials recruiting polyarticular course JIA have excluded other children who may have benefited, for example children with three or four active joints (so-called oligoarthritis or oligoarticular course), or in some trials those with the major comorbidity, JIA uveitis, were also excluded. In real-world practice, many physicians now prescribe biological agents for children with three or four joints involved, especially those with large or ‘high burden’ joint involvement (such as the hip or temporomandibular joint).^17^

Systematic reviews to compare the efficacy of different biological treatments in JIA are challenging, but have concluded that evidence suggests that ILAR categories do not predict response to treatment, but that prevalence of the good responder phenotype to different drugs does vary between JIA subtypes. This further indicates that ILAR categories do not predict response outcomes, and are likely heterogeneous in underlying molecular pathotype or signature.^18^

To date, a biological rationale or specific evidence for cytokine dominance has not driven choice of trial agent for specific categories of JIA. An exception is sJIA, itself more homogeneous than other types of JIA, considered by some to be an autoinflammatory disorder.^19^ In sJIA, the rationale for trials of new agents was built on biological evidence of dominant pathological cytokines, for example, use of soluble IL-6R or IL-1 blockade, which are now recommended in some guidelines as initial monotherapy over use of a csDMARD.^10^ Emerging biomarker studies in sJIA have revealed serum proteins which may predict treatment response. For example those with a high serum IL-18:CXCL9 ratio were more likely to have a good response to IL-1 blockade by canakinumab^20^; however, these have not yet been tested in a prospective biomarker-led trial or validated for routine clinical use.

Across all JIA categories measurement of the serum biomarker S100A8/9 (also known as MRP8/14), which correlates with disease activity (both arthritis and uveitis) has been shown to detect a subgroup of patients who respond well to MTX.^21 22^ This biomarker also has utility in predicting those who have a high risk of flare of arthritis if MTX is withdrawn.^23 24^ Decisions driven by this concentration of this biomarker were built into a recent drug withdrawal study of JIA arthritis: patients in whom biomarker-driven decisions were used had in less time on medication than those in standard care.^25^ However, this generic biomarker, while useful, does not help in choice of specific targeted treatment in JIA or JIA-associated uveitis.

Some trials, where an agent already has proven efficacy in the adult counterpart disease, have recruited only specific types of JIA. An example is the recent trials of IL-17 blockade using secukinumab which included only ERA and PsA categories of JIA, both of which are low-prevalent JIA categories, widely agreed to be the counterpart of the adult spondyloarthritides, AS and PsA, respectively.^26^ These study design decisions are partly driven by regulator guidance. Current recruiting trials of IL-12/23 blockade are also restricted to the PsA JIA ILAR subtype.^27^ However, there is good evidence from studies of JIA synovial inflammation biology that a proportion of children with many types of JIA (including persistent and extended oligoarticular, polyarticular RF- and RF+, PsA, ERA and sJIA) display an early ‘IL-17+pathotype’ among CD4+, CD8+, gd+T cell and innate lymphoid cell populations at the site of inflammation.^28–32^ Furthermore, more recent studies suggest that Th17 cells develop in vitro from JIA blood T cells under polarising conditions, more than healthy controls.^32^ In many of these studies synovial cellular signatures correlate with disease activity, and these cells are plastic at the site of inflammation to become highly pathogenic cells which can then also produce granulocyte macrophage colony-stimulating factor (GM-CSF) and other cytokines.^33–35^ For these patients (who can be identified within many ILAR categories), a biomarker-stratified trial design which would identify those who have a high chance of good response to IL-17 blockade, could be considered.^36^

In parallel with increasing evidence for drug efficacy in JIA has come the drive to move towards a treat to target approach in JIA^37^ to achieve early remission, which is known to translate to better long-term outcomes.^38–40^ However, faced with a child or teenager with active arthritis for which MTX is inadequate to control arthritis, there are currently no validated biomarkers which physicians can use to advise on the optimal choice of one specific biological treatment over others. In practice, choices of drug are essentially ‘trial and error’ and rely largely on historic availability, national practice or insurance funding policies, and local experience, rather than a rationale built on underlying pathobiology of the inflammatory process.

### Current outcomes: the unmet need

Despite these developments in the treatment of JIA, outcomes for young people with JIA treated in the modern era remain disappointing. Many studies show that drug-free remission, though widely agreed to be a key target, is rarely sustained and many young people continue to have multiple periods of active disease.^41–43^ In data from 434 young adults with JIA across Nordic countries with a mean age 24 years, 46% still had active disease, defined by not fulfilling criteria for clinically inactive disease^44^ and 38% were still on DMARDs.^41^ A study of >2000 UK cases receiving biologics, showed that within the 2 years of starting a first biologic as many as 25% switch agent and some reach their third or even fourth biological medication.^45^ In two large US cohorts,^46^ 39%–66% of children had chronically poorly controlled disease, despite previous use of 1–5 biological agents (many prescribed off label), during up to 6 years of disease. Use of long-term immune suppressing treatments which are ineffective brings risks of side effects accompanied by progressive decreases in quality of life and function due to uncontrolled arthritis and associated symptoms. Given current treatment choices are not based on a molecular classification, such a pathway also means that some patients may have missed the ‘window of biological opportunity’ when a specific agent may have been most effective, by the time they are offered it.^47^

Thus, there is a huge unmet need for the identification of subgroups of childhood arthritis based on underlying biological pathotype, allowing innovative ways to do trials, with patient selection and therapy matching to molecular pathotype rather than clinical phenotype alone. Through this we would start to maximise our learning and understanding from every child entered into every trial. Children, families and their health professional teams need such development of robust tools to aid choice of medication, to predict response to treatment, to predict when it is safe to stop treatment without risk of flare, and tools to estimate risk of uveitis. All align to a move towards a ‘precision medicine’ approach, and opportunity to deliver on early, sustained disease remission. In addition, there will be opportunities to propose cross indication trials (so called basket trials),^36 48^ where shared pathology is indicated from biological or mechanistic studies and these could allow the inclusion of debilitating orphan IMIDs in which randomised controlled trials (RCT) are not feasible and where currently there are no licensed drugs for disease control. In this regard, the data gained from large consortia-based studies such as the human cell atlas (https://www.humancellatlas.org/) will allow researchers to identify common disease mechanisms and therapeutic targets that are shared across different diseases and across tissues within the same disease.

### Ways forward to achieve these goals

Several areas where change could accelerate progress towards realisation of precision medicine, via a molecular or cellular nomenclature in childhood arthritis, include:

- Making data accessible and sharing of data to enable replication studies or meta-analyses.
- The routine integration of biospecimens into all clinical trials and observational studies.
- Every child being offered the opportunity to be in research studies or trials.
- Not restricting trials in children to narrow indications, for example, IL-17 and IL-23 blockers to be studied only in PsA-JIA/ERA-JIA.
- Innovative adaptive trial designs in the paediatric IMIDs.
- Encouraging and supporting early career biological and laboratory scientists into the field of IMID research, including childhood arthritis.

### Data generation and sharing to enhance translational discovery

To start to address these unmet needs the UK wide CLUSTER (ChiLdhood arthritis and its associated Uveitis: STratification through Endotypes and mechanism to deliveR benefit) JIA Consortium came together funded by the UK Medical Research Council (MRC) to define biomarkers of response to treatment and start to deliver innovative trial design in JIA and JIA uveitis (https://www.clusterconsortium.org.uk). CLUSTER brings together existing data and samples representing 5400 cases of JIA from cohort studies as well as 2 clinical trials in JIA-associated uveitis to deliver stratified medicine approaches. CLUSTER includes patient and industry partners, as well as multidisciplinary expertise in clinical rheumatology, epidemiology and data sciences, genetics, functional genomics, immunological deep phenotyping, statistics, bioinformatics, and stratified medicine; the CLUSTER community is now also part of the wider UK Consortium with a focus on treatment of IMIDs, IMID-BIO (https://www.gla.ac.uk/research/az/imid/). CLUSTER is generating genetic, transcriptomic, proteomic and deep immunophenotyping data linked to clinical data including response to medication and presence or absence of uveitis. In parallel other large efforts include UCAN (Understanding Childhood Arthritis Network) a federation of networks, which has its focus on translational research in childhood arthritis, with hubs in the Netherlands, Canada and Singapore, which aims to discover genetic, biological and phenotypic markers with diagnostic or prognostic potential in JIA (https://www.ucancandu.com). Harmonisation of standard paediatric sample protocols for use in such studies has been proposed,^49^ and for all studies, detailed metadata and sample provenance recording are vital. These and other similar initiatives will provide resources for validation and replication studies provided data are made accessible and interoperable according to FAIR principles.^50^

Critical to success of these and other initiatives is ensuring that there are enough biological samples with linked clinical data within which to embed this research. JIA and its associated uveitis is a group of rare diseases, and therefore, it is important to consider that research should sit in parallel with clinical care to maximise the potential of data or information from a maximum number of CYP with this condition. If more interventional and observational clinical studies were funded and supported to integrate standardised collection of biospecimens, embedded within the cohort or trial design, this would significantly widen the opportunities for discovery, replication and validation in a diverse and inclusive set of populations. This would also facilitate the opportunity to pool or meta-analyse multiple datasets.

Embedding biological research within larger clinical datasets is not without its challenges despite the many opportunities, particular those such as disease and treatment registries which capture real-world data. Missing data are not uncommon and harmonising data from legacy or differently designed cohort studies is complex, particularly where studies have been established initially for differing purposes (eg, disease outcome, drug safety). We have recently addressed these challenges in CLUSTER to enable us to pool data from four different JIA outcome studies.^51^ To maximise the potential and make every case able to contribute to analyses, careful consideration of research design, particular that which is as closely embedded into direct clinical care, is needed. A minimal internationally agreed core data set, to be collected in all routine clinical care, ideally extracted directly from an electronic medical record into a secure trusted research environment would also speed progress. This has been proposed in another rare paediatric IMID juvenile dermatomyositis.^52^ Governance and permissions of such an approach are needed, but it offers the potential of larger sample sizes as it would reduce the time needed currently to duplicate and report into a study as an additional step. The CAPTURE JIA data set is one such proposal.^53^ An alternative model is of large multicentre registry studies, within which comparative studies of different treatment pathways (with linked biological specimens collected) can be performed.^54^ Within carefully managed clinical trials, clinical data are typically more complete, but these are typically over a short time frame, and where biosample analysis has not been built in as part of the primary study design, valuable insights into the drivers of response or lack of response are not revealed.

### Development of novel tissue cellular and molecular signatures of childhood arthritis

Optimal child-friendly clinical biomarkers for precision medicine would ideally be affordable, widely available and measurable in blood, or even urine. However, it is increasingly recognised that informative biomarkers that relate to pathobiology or predictors of response, may not be readily detectable in blood. This may be due either to power limitations when analysing many 1000s of biomarkers in small cohorts, or to intrinsic fundamental differences between blood and tissue compartments, their proteome, metabolome or transcriptome. Many recent insights into tissue resident, cell pathology within the target tissues of IMIDs have shown that key biological pathways or mechanisms may not be reflected in measurable molecules or cells that are present in blood cells or serum.^55–58^

Historically, many JIA studies used synovial fluid aspirated from inflamed joints of children with JIA undergoing joint injections, as a ‘surrogate’ sampling site which is closer to the diseased tissue than blood. Indeed, synovial fluid inflammatory cells reveal multiple features of the biology that are not detectable in blood, providing clues to underlying molecular and cellular dysregulated pathways, for example, in CD4+Th cells, Treg, B cells dendritic cells and inflammatory mediators. These studies have suggested gene and cellular markers that predict extension in oligoarticular JIA,^59^ shown that regulatory T cells in the synovial fluid correlate with clinical phenotype^60^ but are functionally distinct from healthy Treg.^61 62^ They also identified a ‘pathogenic CD4+T cell’ phenotype that, once defined, can then be identified in peripheral blood,^63^ or revealed that the synovial T cell IL-17 rich signature is present in a proportion of cases across multiple ILAR categories of JIA early in disease.^31^ Recently single cell and epigenetic analyses on synovial fluid cells have further confirmed the many unique features of cells found in the inflamed synovium compared with the homologous cells in blood.^64–66^ However, a limitation of synovial fluid is that it does not include all representative tissue populations, such as stroma and synovial tissue fibroblasts and macrophages, which are thought to play pivotal roles in the balance between tissue inflammation and resolution^57 67 68^

In rheumatoid arthritis (RA), the development of minimally invasive, ultrasound guided synovial biopsy techniques, combined with significant advances in technology, including the ability to perform multimodal cellular and spatial tissue analysis at the single cell level, has led to a paradigm shift in our understanding of tissue-based mechanisms of disease.^69–71^ For example, single cell profiling studies of specific tissue resident, effector cell populations have led to novel insights into the phenotypic and functional diversity of synovial tissue cells, while at the same time identifying novel and tractable therapeutic targets.^72–74^ Furthermore, distinct molecular and cellular signatures have been found to associate with histologically defined synovial tissue pathotypes.^75 76^

The recently reported pathology-driven stratification trial, the R4RA trial, compared response to rituximab to tocilizumab, using a B cell score (defined either by histopathology or whole tissue transcriptome) to test the hypothesis that patients with ‘B cell poor’ tissue would achieve a less good response to B cell depletion and identified specific gene expression signatures associated with good response to either rituximab or tocilizumab, whereas a fibroblast-rich gene signature in the tissue was associated with poor treatment response to all treatments.^77 78^ At the single cell level, multicentre studies led by the Accelerated Medicines Programme consortium funded by the National Institute of Health (URL: https://www.fnih.org/our-programs/AMP/amp-ra-sle), have defined the cellular atlas of the inflamed synovial tissue in RA^79^ and more recently, based on a comprehensive single cell, multimodal analysis of synovial tissue cells proposed six distinct cell-type abundance phenotypes (CTAPs) that reflects the underlying disease heterogeneity.^80^ While it is yet to be determined how these CTAPs associate with disease state or treatment response, it is hoped, that collectively such tissue-based studies, will lead to the development of powerful stratification tools that can be incorporated into predictive algorithms of treatment response and/or used as tissue-based outcome measures of therapeutic response in clinical trials.

While it was previously thought that obtaining synovial tissue biopsies for research purposes would not be possible in children, a recently launched study, powerfully facilitated by the strong support of parents of children with arthritis and patients with JIA themselves, has successfully confirmed the feasibility and tolerability of such an approach in children with JIA.^81^ This opens up the possibility of the cellular and molecular characterisation of disease mechanisms in the target tissues of inflammation in JIA. It is hoped that such an approach will facilitate the development of a molecular tissue-based taxonomy of disease that will underpin biopsy-driven, pathology-led treatment stratification trials in JIA in the future. The MRC-funded Tissue Research in Childhood Arthritis consortium is building capacity within the paediatric research community to perform multicentre synovial tissue biopsy studies in the future across the full spectrum of JIA.

The study of childhood onset monogenic arthropathies, although they are each rare, has provided mechanistic insights which will be invaluable to interrogate such tissue datasets in the future. An example is the study of patients with gain-of-function mutations in NOD2 demonstrating a genetic and molecular phenotype causing Blau syndrome (and a presenting phenotype of early onset sarcoidosis), who may present with tenosynovitis, rash and uveitis.^82^ The protein product of *NOD2* is an innate sensing receptor, which after ligation leads to cytokine production and inflammation through NFkB. NOD2 signalling has been shown to impact Th17 development^83^ and play a role in cytokine production in adult ankylosing spondylitis^84^ and NOD2 has recently been proposed to play the role of ‘rheostat’ in T cells, opening new treatment avenues which may block NOD2 signalling for arthritis.^85^ A further example in patients homozygous for *LACC1* loss-of-function mutations, in which macrophage dysfunction leads to autophagy and altered lipid metabolism in myeloid cells.^86^ This insight has opened potential new therapeutic avenues : recently LACC1 has been shown to play a role in L-ornithine production and treatment of LACC1-deficient mice with L-ornithine was able to reverse inflammation in an infection model.^87^ These insights into monogenic disease mechanisms will help identify new targets in JIA and potentially more common diseases. Such data will also be highly valuable to input into future systems-based analyses across tissues and diseases, and may provide valuable proof-of-principle rationale for cross disease trials, discussed below.

### Innovative trial designs in the paediatric arthritis: the need for clinicians, academia, industry and regulatory bodies to work more cohesively

Several recent initiatives have highlighted the need for a change in how we build the evidence base for personalised medicine in childhood onset arthritis.^46 88^ A report of an international stakeholder meeting including physicians, families, caregivers, industry, charity funders and regulatory agencies provided a clarion call of the need for new ways to perform trials which are more patient-centred and that address this need for precision medicine.^88^ Each phenotype within the group of conditions currently called JIA, is rare. This fact, combined with the increasing availability (where finances allow) of a wide variety of biological agents for treatment make it increasingly challenging to recruit to conventional clinical trials. Trials limited to subsets based on efficacy already determined in phenotypes of adult arthritis, for example, PsA, serve only a small proportion of the total potential beneficiaries for a specific agent. Randomised withdrawal trials using a placebo arm, which hitherto have been the ‘standard design’ are now unpalatable to our patients/parents^88^ and do not answer some of the key clinically relevant questions in the management of children with inflammatory arthritis.

**Figure 1:**
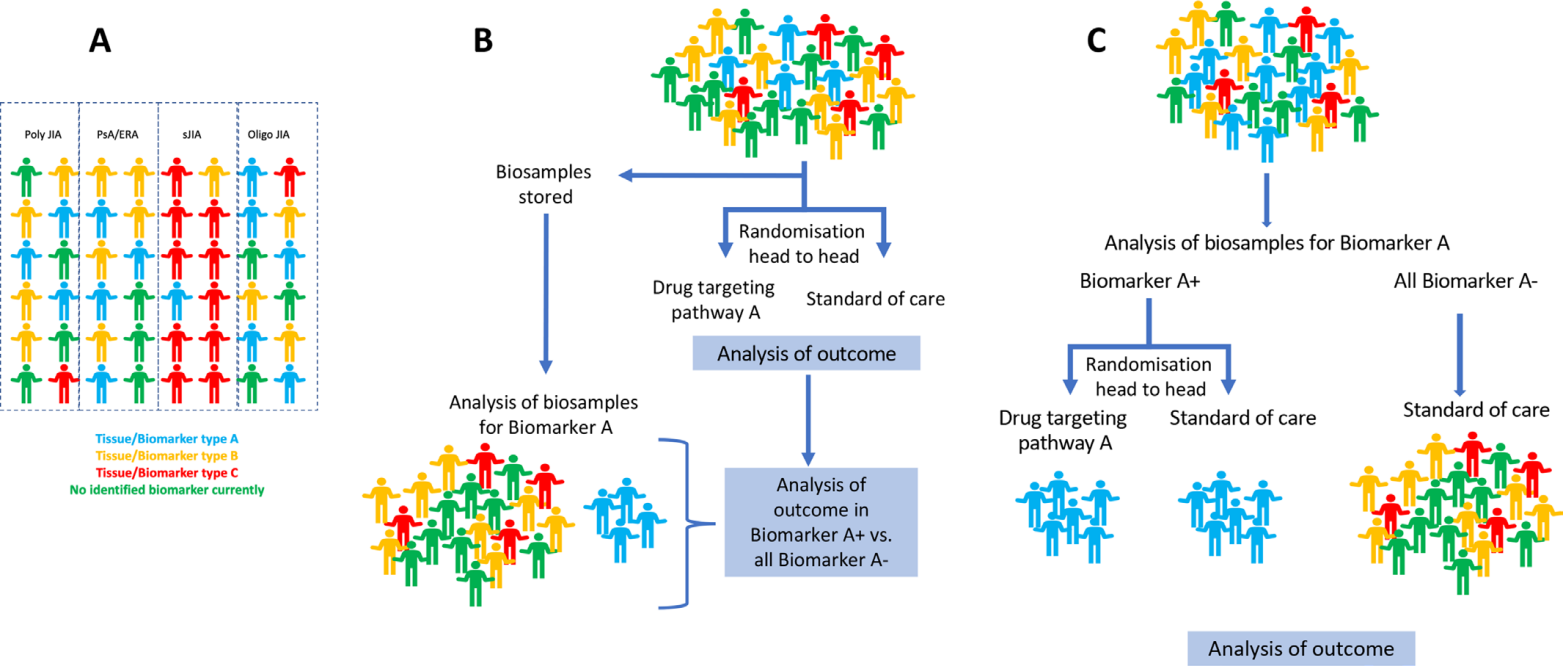
Schematic representation of a biomarker led precision approach in childhood arthritis. (A) Indicates a hypothetical population of 48 children with arthritis currently classified according to ILAR JIA classification criteria, in whom theragnostic biomarkers (represented as A–C in different colours) exist; some are enriched in one ILAR category while others are present in many categories; (B) indicates a trial design where many categories of JIA are included in a trial of a new agent which targets pathway A, compared with a standard of care medication. Biosamples are stored at the start of the trial, but only analysed after study outcome data are complete, to test the hypothesis that biomarker A (blue) associates with a higher rate of good response to the test drug; (C) Biomarker-led design where biomarker A is measured prior to inclusion and only biomarker A+ cases are randomised to drug targeting A, compared with standard of care, so enriching for this group and potentially improving signal. All other cases of arthritis (biomarker A−) are included and given standard of care for comparison. Wedderburn *et al* Annals of Rheum Dis review R1 21.11.22. ILRA, International League of Associations for Rheumatology; JIA, Juvenile idiopathic arthritis.

New trials in childhood arthritis would ideally include head-to-head trials, comparing agents that reflect real-world choices for physicians and families in the clinic. We suggest using adaptive designs with reference arms to enable active comparison with current ‘standard of care’. There are some trials now pioneering this approach.^89^ It is important to encourage industry and regulators to consider novel designs with small sizes to perform meaningful clinical trials.^14^ Novel trial designs will not be unique to paediatric rheumatology: this approach has been advocated or implemented in other fields including oncology and infection.^90 91^ In parallel, regulators should incentivise to ensure that trials are not done in isolated JIA categories but, where appropriate, across all types of childhood arthritis. A vital component in such trials will be the collection of biospecimens to enable biomarkers to be sought and ultimately, molecular pathotypes to be defined. Just as regulatory pressure has led to improved outcomes of children with arthritis through clinical trials, regulatory changes can lead to improvements in trial design and ensure biomarker led clinical trials become the norm. Trials could be required, by funding agencies or regulators, to include biomarker experts as part of trial planning committees, to collect biosamples using robust standardised operating procedures, and to have in place a prespecified experimental and analysis plan for use of samples, as well as where possible, data sharing and open access publication policies. Such data could then contribute to artificial intelligence (AI)-driven integration of large bioinformatic and molecular phenotyping datasets to look for common disease signatures that can be used for treatment stratified precision approaches. A schematic representation of some such trial designs with biomarkers embedded within them is shown in figure 1.

These approaches would also widen study inclusion, to include less common presentations of childhood arthritis or arthropathy which are currently excluded from clinical trials. Chronic non-infectious osteitis (CNO, also known as chronic recurrent multifocal osteomyelitis,) is one such example. Although it is recognised that CNO is part of the psoriatic arthritis/spondyloarthropathy spectrum, there are currently no licensed or approved therapies for this condition.^92^ Therefore, one model would be a single agent industry-led trial, performed in a so called ‘basket trial’ design where several indications are included in the trial for example, IL-17 blocker in extended oligo articular, polyarticular JIA, JPsA, ERA and CNO. Primary endpoints in such a trial design need to incorporate clinically appropriate measures for each condition, but also specific shared biological endpoints assessing the specific molecular target.

A further option is of adaptive or Bayesian design where all prior relevant information is used to inform trial design and far smaller numbers of patients are required. This model has recently successfully been used in the rare paediatric vasculitis condition, childhood polyarteritis nodosa (the MYPAN trial) which demonstrated non-inferiority of MMF compared with cyclophosphamide for induction of remission.^93^ An ongoing multicentre, phase-3 trial of baricitinib in active JIA-associated uveitis or chronic ANA-positive idiopathic uveitis using an open-label Bayesian design will enrol only between 20 and 40 patients.^89^

## Conclusions

The future for paediatric rheumatology is bright since the increasingly large datasets from clinical studies, registries, as well as genetic, omic and tissue studies, combined with international collaboration, family stakeholder involvement and strong relationships with industry partners provide many opportunities for novel routes to discovery and proposals for trials. We suggest that biomarker-driven or biomarker-stratified trials, followed rapidly by biomarker validation in well-curated, longitudinal, real-world registry studies with existing biobanks or available omics data from tissue and blood, may be a feasible and desirable route to achieving truly patient-centred precision medicine for CYP with arthritis.
